# Alcohol Drinking Impacts on Adiposity and Steatotic Liver Disease: Concurrent Effects on Metabolic Pathways and Cardiovascular Risks

**DOI:** 10.1007/s13679-024-00560-5

**Published:** 2024-03-23

**Authors:** Diego Martínez-Urbistondo, Nuria Perez-Diaz-del-Campo, Manuel F. Landecho, J. Alfredo Martínez

**Affiliations:** 1 Departamento de Medicina Interna, Area de Medicina Vascular-Madrid, Clinica Universidad de Navarra, Madrid, Spain; 2 Department of Medical Sciences, University of Turin, Turin, Italy; 3 Obesity and General Health Check-Up Area, Internal Medicine Department, Clínica Universidad de Navarra, Pamplona, Spain; 4 Biomedical Research Networking Center for Physiopathology of Obesity and Nutrition (CIBERobn), Instituto de Salud Carlos III, Madrid, Spain; 5 Precision Nutrition Program, Research Institute on Food and Health Sciences IMDEA Food, CSIC-UAM, Madrid, Spain; 6 Centre of Medicine and Endocrinology, University of Valladolid, Valladolid, Spain

**Keywords:** Alcohol intake, MASLD, Cardiovascular diseases, Fat distribution, Precision medicine, Epidemiological studies

## Abstract

**Purpose of Review:**

This integrative search aimed to provide a scoping overview of the relationships between the benefits and harms of alcohol drinking with cardiovascular events as associated to body fat mass and fatty liver diseases, as well as offering critical insights for precision nutrition research and personalized medicine implementation concerning cardiovascular risk management associated to ethanol consumption.

**Recent Findings:**

Frequent alcohol intake could contribute to a sustained rise in adiposity over time. Body fat distribution patterns (abdominal/gluteus-femoral) and intrahepatic accumulation of lipids have been linked to adverse cardiovascular clinical outcomes depending on ethanol intake. Therefore, there is a need to understand the complex interplay between alcohol consumption, adipose store distribution, metabolic dysfunction-associated steatotic liver disease (MASLD), and cardiovascular events in adult individuals. The current narrative review deals with underconsidered and apparently conflicting benefits concerning the amount of alcohol intake, ranging from abstention to moderation, and highlights the requirements for additional robust methodological studies and trials to interpret undertrained and existing controversies.

**Summary:**

The conclusion of this review emphasizes the need of newer multifaceted clinical approaches for precision medicine implementation, considering epidemiological strategies and pathophysiological mechanistic. Newer investigations and trials should be derived and performed particularly focusing both on alcohol’s objective consequences as putatively mediated by fat deposition, including associated roles in fatty liver disease as well as to differentiate the impact of different levels of alcohol consumption (absence or moderation) concerning cardiovascular risks and accompanying clinical manifestations. Indeed, the threshold for the safe consumption of alcoholic drinks remains to be fully elucidated.

**Graphical Abstract:**

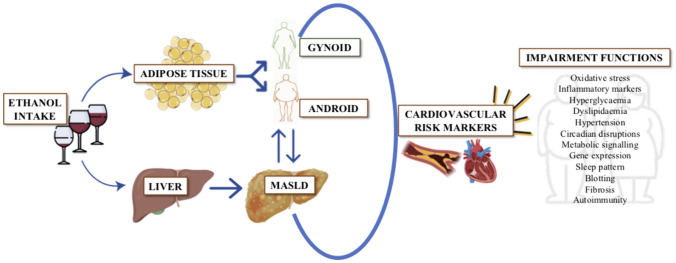

## Introduction

Renewed scientific efforts analyzing the impact of alcohol consumption patterns on health are crucial for disease prevention and management as well as for early management of eventual morbidity risks and for recognizing the role of healthy lifestyles concerning ethanol drinking [1]. Among adult individuals, there is a medical controversy regarding alcohol intake threshold, with current conflicting messages concerning abstention and “moderation,” as well as about unanswered questions concerning physiological benefits and damages attributable to alcoholic beverages [2]. Indeed, abstention is habitually recommended due to alcohol’s overall harmful effects [3–5]; however, moderate intake might be permissible, as evidenced in modest drinkers (both men and women), who showed lower premature mortality, less cardiovascular outcomes, and reduced diabetes or associated health medical risks comparing to abstainers as found in some non-randomized studies [6–8].

To solve these doubts, robust methodological studies such as randomized clinical trials with appropriate controls, are needed [5, 9]. Although these investigations face feasibility and ethical challenges, some research addressing the Mediterranean diet and lifestyle changes including wine drinking have shown that these barriers can be overcome, as evidenced by PREDIMED and PREDIMED plus studies, confirming that such endeavors are possible [10, 11]. However, it is still required to have a precision medicine approach that evaluates not just the epidemiological associations concerning alcohol drinking but also the underlying physiological cues as well as the putative metabolic mechanisms involved [1].

Excessive alcohol consumption is widely recognized as a significant contributing factor to the development of obesity and cirrhosis, both of which pose serious public health burdens [12]. In addition, obesity is associated with serious clinical consequences such as cardiovascular clinical manifestations, chronic metabolic complications, type 2 diabetes, hepatic cancer, and adverse respiratory conditions [13]. In this regard, the relationships between alcohol intake and cardiovascular health depending on factors, such as fat distribution and the occurrence of fatty liver disease, could be a critical concept to understand interactions and effect modifications concerning the health impact of ethanol drinking [14]. The shape of body fat distribution (pear/apple), as well as the amount of intrahepatic fat, are linked to different clinical cardiovascular events and liver injuries, which need to be ascertained [14, 15]. These conditions may be fostered by alcohol drinking, which can affect lipid and mitochondrial metabolism, cause inflammation and unbalanced oxidative stress as well as alter the eubiotic gut microbiota equilibrium, existing evidence that all these factors together contribute to the disease’s overall morbidity and early mortality rates [16].

In this context, there is a need to provide a holistic vision incorporating the integration of findings from recent studies offering a more nuanced understanding about the role of lifestyle factors and genetic predispositions influencing the development and progression of liver disease and associated cardiovascular implications [17–21]. This approach not only highlights the multifaceted impact of alcohol consumption in the context of obesity and liver disease but also underscores the complexity of these conditions, advocating for a multidisciplinary approach to the prevention, diagnosis, and management of cardiovascular disease (CVD) [22]. To this end, literature searches were conducted, including articles describing cross-sectional, cohort (longitudinal), and experimental (interventional) studies, focusing on the impact of alcohol on cardiovascular risk and putative associations with adipose tissue and liver disease, which were initially identified through search strings in multiple databases.

This updated review aims to in-deep analyze interactions concerning alcohol consumption on fat adipose/liver deposition and the subsequent impact on cardiovascular health as well as provide insights that resolve current conceptual uncertainties in both epidemiological and clinical settings, aligning with personalized precision medicine, as well as interventions by clarifying the role of moderate ethanol intake and safe thresholds on health.

## Impact of Ethanol Drinking on Obesity, Metabolic Dysfunction-Associated Steatotic Liver Disease, and Cardiovascular Risks

The relationship between alcohol consumption, obesity, and the risk of developing diseases such as liver disease, including metabolic dysfunction-associated steatotic liver disease (MASLD), is complex and multifaceted [17]. While the precise threshold of alcohol consumption that significantly affects body weight or obesity risk remains under debate [12, 23, 24••], it is clear that alcohol’s caloric content (7 kcal/g) can substantially contribute to daily energy intake. This final energy surplus plays a crucial role in obesity, a condition characterized by excessive or abnormal fat accumulation with demonstrated adverse health outcomes [25]. The interaction between macro/micronutrient intakes and genetic traits further complicates understanding these relationships [25, 26].

Among the different methods for the quantification and estimation of body fat, the DEXA approach is considered objective and sufficiently accurate as the reference method for the measurement of body fat and adipose tissue location; however, the habitual use is limited to the clinical setting and it is difficult to use in population groups [27]. On the other hand, bioelectrical impedance analysis is more accessible and widely used due to inherit low cost and non-invasiveness [28, 29]. A body mass index (BMI) greater than 30 kg/m^2^ [30] remains the most common measurement for diagnosing obesity, due to practical accessibility and affordability, which is associated with morbid events including mortality [31]. However, this adiposity surrogate marker has various limitations as it does not account for body composition or fat distribution, as well as other relevant factors from the health perspective such as sex [32], age [33], or ethnicity [34]. In the last few years, the importance of waist circumference as a risk factor for mortality in older adults as well as other anthropometric and fat distribution approaches has been noted as an independent risk factor for early mortality, emphasizing the role of visceral fat on cardiovascular diseases [15].

Indeed, excess body fat, particularly when located in the visceral compartment and associated metabolic impairments significantly increased cardiovascular disease risk [35], along with other clinical manifestations such as diabetes [26], hypertension [36], dyslipidemia [37•], heart failure [38], or MASLD [39], among other morbid complications [25].

Alcohol abuse and unhealthy dietary patterns contribute to liver disease, by promoting an inflammatory and oxidative stress environmental imbalance that favors hepatic fibrogenesis [40]. In this regard, a study with a median follow-up of 4.4 years showed that a larger visceral adipose tissue area at baseline was positively associated with a higher incidence of MASLD in a dose-dependent manner (HR, 2.23; 95% CI, 1.28–3.89) [41]. In addition, a cross-sectional study from NHANES 2017–2018 reported that a higher android/gynoid ratio (A/G ratio) was linked with a higher prevalence of MASLD in both men (OR, 1. 79; *P* = 0.029) and women (OR, 1.95; *P* = 0.023). Concerning hepatic fibrosis, the A/G ratio was positively associated in women (OR, 2.09; *P* = 0.026) and was marginally inversely in men (OR, 0.56; *P* = 0.078) [42]. Noteworthy, weight loss (even modest) reduces MASLD progression and benefits clinical markers and hepatic health indicators or intrahepatic fat deposition [43]. This connection underscores the importance of considering alcohol intake in the context of liver health and cardiovascular risk.

Moreover, the new definition of steatotic liver disease have differentiated between MASLD, with moderate consumption and cardiometabolic risk factors vs. Met-ALD (metabolic dysfunction and alcohol-related steatotic liver disease) when alcoholic intake exceeds the terms of moderation, highlighting the nuanced relationship between alcohol, liver health, and cardiovascular outcomes [44••]. Observational studies suggest that the liver’s condition modifies alcohol’s health effects, affecting cardiovascular morbidity and associated deaths [14]. In this regard, low-moderate alcohol consumption decreased all-cause mortality and CVD risk, but only among non-smokers, whereas drinking more than an average of one and a half drinks per day was associated with an increase in mortality in patients with MASLD [14, 45]. Indeed, patients with MASLD could seemingly benefit more from moderate alcohol consumption concerning cardiovascular protection than patients without excess hepatic fat according to an available cohort study [45]. However, the combined prevalence of metabolic syndrome and alcoholic consumption appears to evidence deleterious consequences on hepatic and overall disease progression as well as on global morbi-mortality rates [46–48]. A recent study stated that the evidence was limited or inadequate to demonstrate that alcohol reduction or cessation reduces breast or liver cancer risk [49].

The interplay between alcohol, obesity, and liver disease exacerbates cardiovascular risk through various biological mechanisms [39], including liver dysfunction, inflammation, increased arterial plaque deposition, heightened blood pressure, and altered lipid profiles [39, 50]. This combination of factors suggests a synergistic effect, where MASLD, alcohol, and obesity significantly influence cardiovascular health. In this regard, numerous studies and trials have explored the impact of ethanol drinking on obesity, liver diseases, and cardiovascular risk (Table 1). Furthermore, emerging research, including Mendelian randomization studies, that screened the mediating role of genetic predisposition in the development of disease secondary to the confluence of.

**Table 1:** Selection of studies and trials in chronological order concerning the influence of ethanol intake on obesity, MASLD, and cardiovascular risk

**Author, year**	**Study design**	**Hypothesis/aim**	**Main variables**	**Outcomes/major findings**
John B. Dixon et al. 2002 [51]	Cross-sectional study with 486 severely obese subjects	To examine the association between the clinical and biochemical features of the metabolic syndrome and quantity and type of alcohol intake in the severely obese	Adipose tissue (BMI)	Light-to-moderate alcohol consumption is associated with a lower prevalence of type 2 diabetes, reduced insulin resistance, and a more favorable vascular risk profile in the severely obese
Rosalind A. Breslow et al. 2005 [52]	Cross-sectional study 45,896 adults, never smokers who were current alcohol drinkers	Examined the relation between drinking patterns and BMI	Adipose tissue (BMI)	Participants consuming the smallest quantity the most frequently were leanest, and those who consumed the greatest quantity the least frequently were heaviest. Alcohol may contribute to excess body weight among certain drinkers
Mattias Ekstedt et al. 2009 [53]	Longitudinal study 71 patients with chronically elevated liver enzymes and diagnosed with biopsy-proven MASLD	To investigate whether low alcohol intake, consistent with the diagnosis of MASLD, is associated with fibrosis progression in established MASLD	Liver disease (MASLD)	Moderate alcohol consumption, consistent with the diagnosis of MASLD to be set, is associated with fibrosis progression in MASLD
Carole L. Hart et al. 2010 [54]	RCT longitudinal Midspan prospective cohort studies (*n* = 9559 men)	To investigate whether alcohol consumption and raised BMI act together to increase the risk of liver disease	Adipose tissue (BMI) and liver disease (liver diseases and liver cancer)	Raised BMI and alcohol consumption are both related to liver disease, with evidence of a supra-additive interaction between the two
Rohit Loomba et al. 2010 [55]	RCT longitudinal 2260 Taiwanese men from the Risk Evaluation of Viral Load Elevation and Associated Liver Disease/Cancer-Hepatitis B Virus Study Cohort	To determine if body mass index and alcohol use have synergistic effects on HCC risk	Adipose tissue (BMI) and liver disease (HCC) markers	The risk of incident HCC increased in overweight (HR, 2.4; 95% CI, 1.3–4.4); obese (HR, 2.0; 95% CI, 1.1–3.7); and extremely obese (HR, 2.9; 95% CI, 1.0–8.0) users of alcohol (*P* for trend = 0.046) in hepatitis B surface antigen-positive men
Simona Costanzo et al. 2010 [56]	Meta-analysis 16,351 patients with cardiovascular disease	To quantify the relation between alcohol consumption and cardiovascular and total mortality in patients with a history of cardiovascular events	Cardiovascular disease markers	In patients with cardiovascular disease, light-to-moderate alcohol consumption (5 to 25 g/day) was significantly associated with a lower incidence of cardiovascular and all-cause mortality
Carmen Sayon–Orea et al. 2011 [57]	Longitudinal study 9318 adults without previous chronic disease	To evaluate the association between the type of alcoholic beverage intake and weight change in a Mediterranean cohort	Adipose tissue (BMI)	The type of alcoholic beverage can modulate the effect of alcohol intake on the risk of developing overweight/obesity
Kayoung Lee et al. 2012 [58]	Cross-sectional study 3793 (963 men and 1830 women) current drinkers	To examine gender-specific relationships between alcohol drinking patterns (frequency, typical drinking quantity, and frequency of binge drinking) and the prevalence of metabolic syndrome and its components	Cardiovascular markers	Binge drinking frequency was dose-dependently associated with high TAG, high glucose, high blood pressure, and abdominal obesity in men, and with high glucose and high blood pressure in women. Average drinking frequency was not associated with the prevalence of MetS in either sex
Cosmin S. Voican et al. 2015 [59]	Longitudinal study 47 patients with ALD were prospectively included	To study the consequences of alcohol withdrawal in macrophage markers and polarization in the SAT of alcoholic patients and adipokine expression according to liver inflammation	Adipose tissue (inflammatory biomarkers)	One week of alcohol withdrawal alleviates macrophage infiltration in subcutaneous adipose tissue and orients adipose tissue macrophages towards a M2 anti-inflammatory phenotype; this implicates alcohol in adipose tissue inflammation
Michael Roerecke et al. 2019 [60]	Systematic Review and Meta-Analysis 2,629,272 participants with 5505 cases of liver cirrhosis	To systematically summarize the risk relationship between different levels of alcohol consumption and incidence of liver cirrhosis	Liver disease (cirrhosis)	There was no increased risk for occasional drinkers. Consumption of one drink per day in comparison to long-term abstainers showed an increased risk for liver cirrhosis in women, but not in men
Elif Inan–Eroglu et al. 2022 [61]	RCT longitudinal 465,437 participants from the UK Biobank	To examine the associations of adiposity and alcohol consumption on ALD, MASLD, and liver disease incidence and mortality	Adipose tissue (BMI and WC) and liver disease (MASLD and ALD)	Overweight/obese participants with alcohol consumption above the guidelines had a greater HR for liver disease incidence and mortality (HR 1.52 and HR 2.20, respectively) than normal weight individuals (HR 0.95 and HR 1.24, respectively)
Briansó Llort L. et al. 2022 [62]	RCT and cross-over 29 healthy male and female (45–75 years)	To analyze if resveratrol content in red wine increases SHBG levels	Cardiovascular disease	Red wine rich in resveratrol reduces total cholesterol in men and women and increases SHBG only in women
Laurens A. van Kleef et al. 2023 [63]	Longitudinal study 12,656 participants NANHES cohort	To study the mortality risk of MASLD in relation to excessive alcohol consumption and its potential interactions	Liver disease (MASLD)	Participants with both MASLD and excessive alcohol consumption expressed the highest mortality risk (aHR, 1.47; 95% CI, 1.28–1.71)

*ALD* alcoholic fatty liver disease, *BMI* body mass index, *HCC* hepatocellular carcinoma, TAG triacylglycerol, *MASLD* metabolic dysfunction-associated steatotic liver disease, *SAT* subcutaneous adipose tissue, *WC* waist circumference

MASLD and alcohol consumption, underscored the need for further in-depth research of the alcohol-fatty liver binomial axis in order to establish solid evidences [64, 65]. Indeed, excess energy supplied, provided by alcohol intake, may influence fat deposition and distribution (visceral vs. gluteus-femoral or intrahepatic lipid), being affected by sex, which may further impact cardiovascular risk [66, 67]. This effect has been observed across different patterns of alcohol consumption, which influence central adiposity, and showed that the frequency and intensity of alcohol intake have different effects on fat which is associated with cardiovascular risk [66]. In particular, the role of alcohol in elevating plasma androgen levels remains significant even when controlling for factors such as BMI, pointing to its direct contribution to androgenic fat accumulation [68]. Moreover, sex-specific effects are evident in the way alcohol consumption affects liver health and metabolic regulation, particularly in men with MASLD who consume more than 70 g of alcohol per week, underscoring the nuanced relationship between alcohol, fat distribution, and cardiovascular risk [67].

## Complexity of Evaluating the Impact of Alcohol on MASLD

The relationship between alcohol consumption and MASLD is complex due to the underlined confluence of multiple risk factors, heterogeneous clinical determinants, fat mass adaptive pathophysiological mechanisms, and medical outcomes diversity [69]. These interactive features could partially explain the inconsistencies among different observational studies and justify the need for mechanistic studies based on randomized intervention trials concerning alcoholic consumption advice (abstinence vs. moderate consumption) despite some practical and ethical constraints [14, 45, 70]. In this context, to understand the underlying mechanisms of the MASLD-alcohol interaction and generate impartial results, novel trials should evaluate the longitudinal influence of some related factors and determinants. This includes the objective measurement of alcohol consumption, the standardized evaluation of lifestyle as well as genetic predisposition screening, and the assessment of cardiovascular risks and hepatic markers in terms of fatty liver, steatohepatitis, and hepatic fibrosis. Additionally, the studies should consider the occurrence of robust clinical cardiometabolic outcomes, as well as the progression and validation of surrogate indices of disease [18].

The objective assessment of alcohol consumption is a major challenge in studies assessing metabolic impacts on health [71•]. The inaccuracy in reporting alcohol intake, recall bias, “sick quitter” bias, and both voluntary and involuntary underestimation of consumption can invalidate the results [72]. This lack of rigor is even more significant in MASLD studies, where misclassification of patients around 30% has been detected [72]. Among the potential markers of alcohol consumption, urinary and hair ethyl glucuronide has shown a remarkable ability to objectively define alcohol consumption, with a discriminatory capacity of these patients above 90% [72]. Furthermore, other biomarkers such as cytokeratin 18, elevated liver enzymes, serum bilirubin, or elevated serum white blood cell count have been suggested to have prognostic value in alcohol-related diseases [73].

Exposome data and lifestyle evaluations are crucial in patients with metabolic risk and fatty liver disease [74]. In this context, weight loss has been linked to a reduction in hepatic fat and comorbidity in patients with liver condition [75]. The Mediterranean diet pattern has shown a positive impact on both hepatic fat and cardiovascular risk despite a high lipid intake [75, 76•]. Similar benefits apply concerning exercising and physical activity practice [77]. Genetic predisposition plays a role in the onset, progression, and extrahepatic complications of fatty liver disease, whether alcoholic, metabolic, or mixed causes could be the physiopathological trigger [78]. In this context, single nucleotide polymorphisms (SNPs) in genes such as *PNPLA3*, *MBOAT7*, *APOE*, and *TM6SF2*, with mutation rates between 7 and 37%, are linked to the progression of liver disease due to excess fat of metabolic and alcoholic origin [79]. Thus, the different phenotypic manifestations and severity of MASLD are the outcome of complex traits influenced by the interaction of genetics, nutrient intake/exposure, and environment and behavioral factors [69]. In this regard, a predictive model based on 22 SNPs related to obesity and weight loss allowed personalization of the most appropriate diet for 72% of MASLD patients [80]. The relationship between genetics and liver disease is of particular interest in the cardiovascular field due to the results of Mendelian randomization studies, where genes predisposing to the establishment of excess hepatic fat through reduced efflux of very low density lipoprotein (VLDL) particles into the bloodstream showed no association with increased cardiovascular risk, while the assessment of other SNPs related to elevated liver enzymes, the presence of hepatic fat in ultrasound, and MASLD confirmed by biopsy does allow for establishing this relationship as causal, which need to be analyzed concerning alcohol drinking interactions and levels [79].

## Holistic Cardiometabolic Risk Management: Impact of Ethanol Consumption on the Adipose-Liver Interrelationship

An accurate clinical examination is important for understanding etiological and mediating factors in field studies concerning alcohol drinking and associated clinical interactions [81, 82]. In this context, the quantification of cardiovascular risk in individuals with putative cardiometabolic impairments for medical intervention is based on the diagnosis of active smoking, hypertension, and dyslipidemia [81]. The 2021 European Society of Cardiology cardiovascular prevention guidelines present risk stratification scales such as SCORE2, which categorize individuals, based on age, sex, and untreated risk factors, a probability of developing cardiovascular events more precise than the sum of factors [83]. However, the existing discrimination ability of these scales is around 70% [82]. Therefore, identifying residual risk factors of (cardio)vascular disease risk is justified. Among these, MASLD could be considered a potential condition of interest [84••]. The liver is central in controlling lipid and glycemic metabolism, as well as in regulating inflammation mechanisms, which are all linked to vascular disease [85, 86, 87•]. Briefly, weight gain triggers adipose tissue expansion and macrophage recruitment through the secretion of various chemokines and cytokines [88]. Inflamed and dysfunctional adipose tissue actively releases free fatty acids into the bloodstream, promotes lipotoxicity in the liver, muscle, and pancreas, and contributes to systemic inflammation [89]. When the hepatic capacity to handle carbohydrates and fatty acids is overwhelmed, triglycerides and a variety of lipid metabolites accumulate in the liver inducing mitochondrial dysfunctions and oxidative stress, unbalanced reactive oxygen species production, and endoplasmic reticulum stimulation, leading to hepatocyte injury, resulting in fibrogenesis and genomic instability that favors the development of more advanced stages of the disease, hepatocarcinoma and death [88]. Additionally, this metabolic disruption can shift the liver’s energy metabolism homeostasis towards ketogenesis, a state where the liver produces ketone bodies for energy [90]. Concurrently, the altered lipid metabolism can contribute to atherosclerosis by promoting the buildup of plaques in the arteries, further complicating the disease’s progression [88]. Concretely, some subjects will develop MASLD, which could lead to metabolic dysfunction-associated steatohepatitis, but others will directly present inflammation and fibrosis, probably because of the influence and interaction of environmental, metabolism, gut microbiome, genetic, and epigenetic factors [91]. Thus, the hyperinsulinaemic, hyperglycaemic, and hypertriglyceridaemic dysmetabolic state of visceral obesity can be partly explained by an elevated hepatic fat content, without a specific contribution from visceral adipose tissue [92], where ethanol role needs to be addressed. For instance, a longitudinal, observational cohort study concluded that hepatic fat was associated with poor cardiovascular health and was linked over time to adverse changes in fasting blood sugar and triglyceride levels, also after considering changes in BMI [93]. Some controversy has risen according to multicentric studies with some investigations defending a very limited role for MASLD in (cardio)vascular disease risk [94], while others support a potential role as a residual risk biomarker [39].

The stratification of fatty liver disease also plays a role in patient prognosis [95•]. Both simple steatosis and steatohepatitis have been shown to influence the overall outcome of patients, although hepatic fibrosis is the marker most associated with morbidity in this population [96]. Unfortunately, for a disease of this relatively high prevalence, hepatic biopsy, an invasive procedure with its own complications, is the standard for diagnosis [97]. However, the development of non-invasive tests such as ultrasound and hepatic elastography avoids the inherent risks associated with invasive measurement, with little loss in discriminatory capacity, although they remain examiner-dependent and have low availability for screening at-risk populations. Therefore, blood markers that allow the assessment of patients with MASLD, such as the Fatty Liver Index or the Hepatic Steatosis Index [98, 99], those with steatohepatitis such as the OWL Metabolomics^©^ lipid diversity interpretation kits [100], and the degree of fibrosis via the Fibrosis Index-4 [101] have demonstrated valid prognostic capability in the general population in prospective cohorts but need to assess the impact and mediation of the amount of alcohol consumption in these processes [95•].

## Factual Considerations on Ethanol Drinking and Disease Markers

Alcoholic beverage consumption has been often related to unhealthy nutritional patterns with impacts on weight gain and liver diseases, but moderate drinking of alcohol has been shown as a component of beneficial dietary patterns in many studies [102]. In any case, alcohol cues may play a role in obesity onset and prevalence by disturbing the endocrine regulation and affecting the neurobiology of feeding behaviors and energy homeostasis, which may demand psychosocial approaches and multidimensional interventions [103•].

Actually, despite the recognized caloric contribution of alcohol (5.6 kcal/mL) to the energy balance, a number of prospective trials have demonstrated that recreational light-to-moderate alcohol intake is not associated with weight gain, while heavy alcohol drinking has been consistently related to obesity [24••, 102]. Furthermore, some evidence have even disclosed that some individuals who habitually drink moderate amounts of alcohol may be protected against obesity and liver steatosis [24••]. However, observational and intervention studies with different cross-sectional and longitudinal approaches or experimental hypotheses including diverse cohorts, in many regions or cultures and age/sex groups have reported contradictory findings, which merits further analyses. Thus, short-term effects of alcohol intake may influence appetite control and drive feeding conducts in humans as well as modulate satiety hormone actions via peptide YY (PYY), ghrelin, gastric inhibitory peptide (GIP), or cholecystokinin (CCK) or food/drink ingestion control through several central neuroendocrinological circuits, while other, potential mechanisms associating ethanol beverages and obesity include binge drinking and the effects of alcohol on opioid, serotonergic, and GABAergic pathways in the brain by increasing appetite as summarized by Traversy and Chaput [24••] and others [103•].

Furthermore, alcohol abuse has an impact on global body health and may contribute to organ and tissue injuries, premature mortality, and the burden of death, with more than 40 International Classification of Diseases categories attributable to high alcohol consumption including hemorrhages, pancreatitis cirrhosis, hepatitis, and ascites, as well as cancer but with curvilinear exceptions involving ischemic diseases and diabetes and with eventual beneficial effects of light drinking in diverse cardiovascular events, whose residual pathophysiological mechanisms need to be elucidated in most cases [104].

Alcohol addicts can primarily experience effects on the brain, varying between diverse neurological and psychological disorders including seizures, ataxia, aggression/violence, social anxiety, and schizophrenia [105•]. Also, these authors collated that cAMP protein kinases, protein kinase C serine-threonine kinases, RACK 1, ERK, and Homer 2 alterations could be part of metabolically stressed pathways by alcohol intake, while ethanol drinking would be linked to epigenetic changes and microbiota dysbiosis [105•]. Furthermore, published studies seem to associate a low-moderate alcohol consumption with lower risks of suffering neurodegenerative diseases, where a J-shaped relationship between alcohol consumption and all-cause mortality was found [106]. A reduced risk for moderate alcohol consumers than for abusive drinkers or abstainers has been evidenced, where a role for polyphenols supply has been mentioned that may explain healthier responses from beers and wine but not spirits use [107].

A chronic high ethanol consumption is a determinant for the development of liver diseases, where oxidative stress being put forward as an important contributor, despite that moderate consumption of red wine has been associated with hepatoprotective effects, mainly due to the antioxidant effect of resveratrol, and other commonly wine-occurring polyphenolic compounds, which can modulate redox signaling [108]. Thus, alcohol consumption constitutes a major factor of morbidity and early mortality, with adverse medical implications that almost affect every cell, including not only the staged alcohol-related fatty liver, alcohol-related steatohepatitis, fibrosis, cirrhosis, and hepatocellular carcinoma but also acute and chronic pancreas dysfunctions, endocrine system and hematological disruptions, renal disease, and urological pathologies; and even infectious diseases are related to alcohol consumption as well as some tumors [109•].

Moreover, alcohol consumption seems to specifically promote abdominal (android) fat distribution, although the role of ethanol in lean subjects is unclear [110••], while alcoholic drinks have been associated with higher magnetic resonance-derived liver fat in an asymptomatic study population [111]. Noteworthy, an abdominal adipose accumulative pattern accompanies increased rates of MASLD in both sexes [42]. However, another trial has shown the lowest odds for hepatic fat fractions in moderate alcoholic drinkers but higher liver lipid accumulation in abstainers or heavy drinkers [111]. Inhibitory actions of ethanol on oxygen uptake, gluconeogenesis, and ketogenesis were investigated in rat livers, where the net effects of ethanol appeared as an ensemble of fractional effects along the hepatic acini [112]. Also, alcohol consumption has been associated in a meta-analysis using polysomnography assessments with sleep disturbances and with apnea-hypopnea index alterations as well as lowest oxygen saturation among patients susceptible to obstructive sleep apnea [113].

Noteworthy, a comprehensive review concerning the clinical impact of alcohol intake on cardiovascular outcomes considering diverse methodologies revealed a cardio-protective relationship between moderate intake of alcoholic beverages and cardiovascular events, which involved the consumption of wines, beers, and spirits or alcoholic cocktails [110••]. Indeed, alcoholic beverages drinking may benefit the circulating lipid profile by rising plasma high-density lipoprotein-cholesterol levels and inhibiting thrombogenesis affecting thromboxane formation and decreasing the plasma level of fibrinogen. However, high blood concentrations of alcohol may impair fibrinolysis by increasing plasma plasminogen activator inhibitor-1 level. These mechanisms could contribute to explaining the “U”-shaped association between alcohol intake and cardiac events [110••].

Wine in particular, but also beer, contains polyphenols which may act as antioxidants and contribute to the integrity of the endothelial function by reducing superoxide production [107]. Such antioxidant molecules may shield against low-density lipoprotein oxidation and temper the macrophage harm on the endothelium. Although the cardio-protective effect of alcohol can barely be analyzed in healthy individuals by interventions with hard end-points, due to ethical arguments there are a number of findings demonstrating that moderate alcohol drinking elicits cardio-preventive functions [110••]. On the other hand, a high alcohol intake is associated with hypertriglyceridemia attributable to a higher VLDL secretion, disturbed lipolysis, and elevated free fatty acid flows from adipose tissue to the liver, but light-to-moderate alcohol consumption may decline circulating triglycerides [114]. Unique risk factors for CVD exist in women, which should be separately addressed including differences in ethanol consumption [115]. A modest alcohol consumption induced a favorable outcome concerning carotid plaque formation or carotid artery stenosis in men with MASLD [116], while a long-term randomized controlled trial found that introducing a moderate red wine intake, among diabetics within a healthy care prescription was secure and moderately reduced cardiometabolic risk [117].

Some benefits of low-to-moderate amounts of alcohol intake compared with abstinence have been also reported concerning longer life expectancy as mediated by inflammation, immunocompetence, and insulin sensitivity modulation regarding intermediate end-points of coronary heart disease, which have has been repeatedly reported from both animal studies and human clinical trials [112, 118•].

**Fig. 1:**
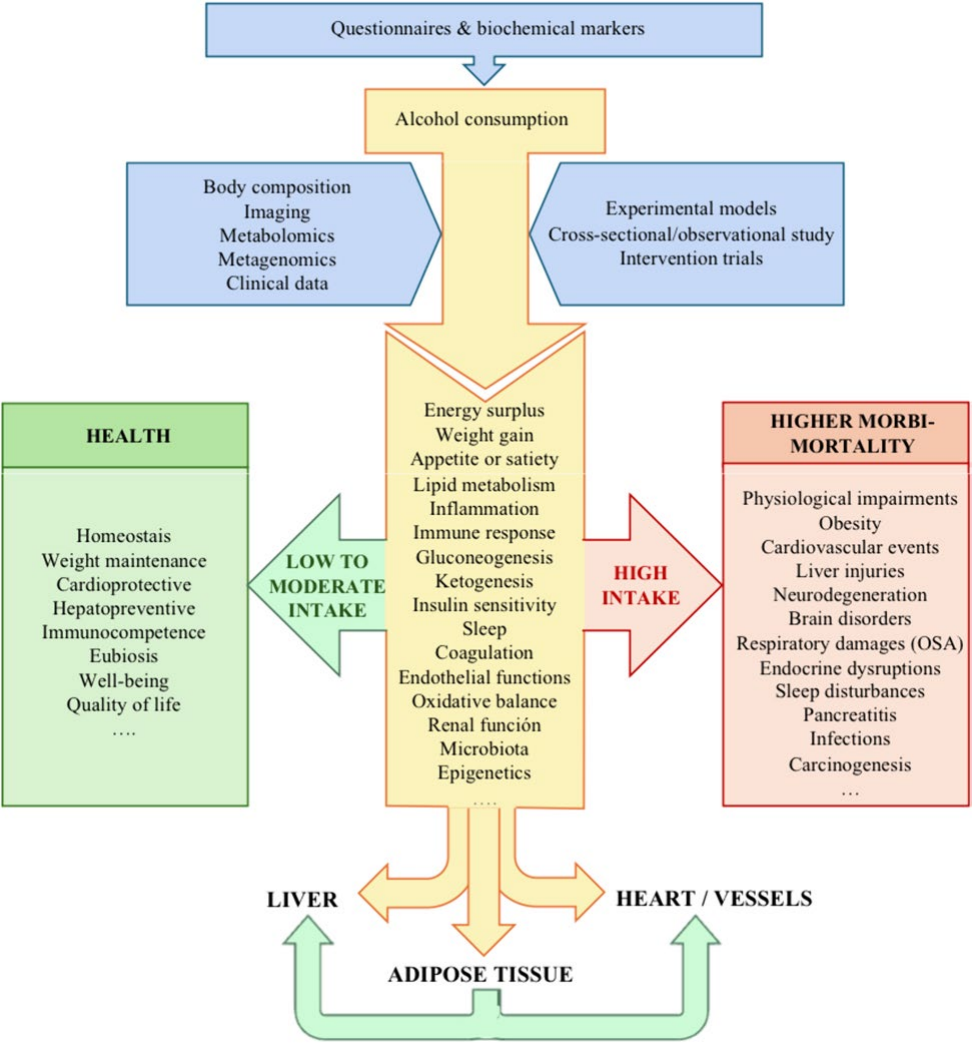
Mechanistic insights for precision medicine implementation: interplay between alcohol intake with obesity, MASLD, alcohol intake, and cardiovascular health. Abbreviations: MASLD Metabolic associated dysfunction steatotic liver disease, OSAS obstructive sleep apnoea

This current review provides a deep analytical literature search pertaining to the impact of alcohol intake on obesity, liver fat-derived diseases, and cardiovascular events considering putative and recognized determinants, participatory mechanisms, and risk factors. Experimental studies and clinical observational cross-sectional and prospective interventions as well as epidemiological investigations have disclosed an inverse correlation between moderate intake of alcoholic beverages and cardiovascular disease. The causal relationship appears to occur for both wine and beer but the effects of alcohol itself and also the role of different cardio-protective substances in alcoholic drinks such as polyphenols need to be further investigated for precision personalized medicine implementation as well as to discern the healthy alcohol thresholds ranging from abstemious to moderate consumers (Fig. 1).

The recruitment of sufficiently large, well-documented cohorts with adequate follow-up is essential to capture robust outcomes such as the incidence of diabetes mellitus, major cardiovascular events, cancer, or overall mortality [9]. The use of appropriate surrogate disease markers could contribute to understand the natural history and the impact of the alcoholic intervention on patients [119]. In this regard, cardiovascular-cause death is the most common cause of premature death in the general population and in patients with fatty liver disease [39, 120••]. For this reason, the longitudinal evaluation of validated proxies included in clinical guidelines for cardiovascular disease, such as coronary calcium, could be useful in monitoring patients undergoing interventions on alcohol intake [121, 122].

Indeed, examining the impact of excessive adiposity and fat distribution (in adipocytes/hepatocytes) related to ethanol intake needs to take sex-related differences into account to better understand the role of ethanol drinking in cardiovascular risk and events, as well as the involved pathophysiological mechanism.

## Conclusions

The effects of ethanol intake affecting fat distribution and hepatic state are relevant questions to be revisited with randomized clinical trials. Furthermore, the integrated evaluation of the impact of alcohol consumption, including the lifestyle assessment, in-depth cardiovascular phenotype examinations, and hepatic clinical stratification, together with considering genetic predisposition and gut microbiota composition is required to understand the physiopathology associated to alcoholic drinking.

A holistic analysis should provide a more precise scope of the health benefits and harms as well as associated morbid mechanisms related to alcohol intake. To conform personalized treatment strategies with an objective comprehension of the different impacts of the level (abstention/moderate vs. heavy) of ethanol intake is crucial for human achieving wellbeing and health. Indeed, the threshold for a safe/healthy ethanol drinking needs to be elucidated, given that there are evidences that moderate ethanol intake may have some modest health benefits, while alcohol abuse is accompanied by devastating effects on cell machinery (inflammation, oxidative stress, immunocompetence, lipid metabolism, etc.), as well as inducing body and diverse organ physiological disruptions. Additionally, tracking validated markers for cardiovascular disease progression may contribute to personalized medicine development, including objective measurement of alcohol intake and omics-based technologies and machine learning tools. Indeed, the safe threshold concerning alcohol consumption needs to be defined with carefully designed intervention studies.

## Data Availability

No datasets were generated or analysed during the current study.

## Abbreviations

ALD: Alcoholic fatty liver disease
BMI: Body mass index
CVD: Cardiovascular disease
HCC: Hepatocellular carcinoma
MASLD: Metabolic dysfunction-associated steatotic liver disease
Met-ALD: Metabolic dysfunction and alcohol-related steatotic liver disease
SNPs: Single nucleotide polymorphisms
VLDL: Very low density lipoprotein
WC: Waist circumference
